# The role and mechanism of irisin/NLRP3 signal in aerobic exercise ameliorating blood glucose homeostasis in pre-diabetic mice

**DOI:** 10.1371/journal.pone.0336395

**Published:** 2025-11-20

**Authors:** Shujuan Hu, Zhengkang Wu, Xuan Liu, Yiting Ding, Jun Chen, Xianwang Wang

**Affiliations:** 1 School of Education and Physical Education, Yangtze University, Jingzhou, Hubei, China; 2 Department of Biochemistry and Molecular Biology, Center for Molecular Medicine, Health Science Center, Yangtze University, Jingzhou, Hubei, China; 3 Shannan Maternal and Child Health Hospital, Shannan, Xizang, China; University of Diyala College of Medicine, IRAQ

## Abstract

Pre-diabetic mellitus (PDM) is characterized by chronic low-grade inflammation, primarily driven by NLRP3 inflammasome hyperactivation and a concurrent deficiency of the myokine irisin. MCC950 is a highly specific inhibitor of the NLRP3 inflammasome, and aerobic exercise has also been shown to effectively suppress its activation. However, the underlying molecular mechanisms remain unclear. The aim of this project was to explore whether the irisin/NLRP3 signaling pathway was regulated by aerobic exercise in mice with PDM. Forty mice were divided into: the common diet group (DC group, N = 10), and the high-fat diet group (HFD group, N = 30). The HFD group received a high-fat diet combined with a single low-dose streptozotocin (STZ) injection to induce a pre-diabetic state. Successfully modeled mice were identified as PDM mice and randomly assigned into three subgroups: the PDM control group (PDM-DC group, N = 8), the PDM plus exercise group (PDM-EX group, N = 8), and the PDM plus MCC950 group (PDM-MC group, N = 8). The PDM-EX group performed treadmill exercise for 4 weeks (5 days/week, 12 m/min, 60 min/d). The PDM-MC group received NLRP3 inhibitor injections (MCC950, 10 mg/kg, 5 d/week) for 4 weeks. These results found that aerobic exercise and MCC950 ameliorated glycolipid metabolism, reduced insulin levels, and effectively facilitated the skeletal muscle remodeling in PDM mice. Compared with the DC group, PDM mice exhibited significantly downregulated *FNDC5*/irisin expression and upregulated NLRP3 and IL-18 expression (*P* < 0.05 or *P* < 0.01). Notably, aerobic exercise significantly increased *FNDC5*/irisin expression (*P* < 0.05), and decreased NLRP3 and IL-18 levels (*P* < 0.01). Cell experiments revealed that the mRNA and protein expression of NLRP3, IL-1β and IL-18 in the high glucose (HG) condition were higher compared with the lower glucose (CON) condition (*P* < 0.01). Treatment with irisin significantly attenuated these increases (*P* < 0.05 or *P* < 0.01). These findings demonstrate that aerobic exercise alleviates inflammation and ameliorates glycolipid metabolism in PDM mice by modulating the irisin/NLRP3 signaling pathway. Moreover, irisin effectively suppresses high glucose-induced upregulation of NLRP3, IL-1β, and IL-18, suggesting its potential therapeutic role in managing pre-diabetic inflammation.

## Introduction

Pre-diabetes mellitus (PDM), or impaired glucose regulation, is a critical stage between normal glucose homeostasis and type 2 diabetes mellitus (T2DM). During this compensatory period, fasting or postprandial glucose levels are only slightly elevated, yet the risk of developing overt diabetes within 5–10 years is approximately 70% [1]. The main characteristic of prediabetes is a certain degree of abnormal glucose and lipid metabolism. The reduction of glucose utilization and the increase of gluconeogenesis are significant factors contributing to the disorder of glucose metabolism [2]. Abnormal increase of total cholesterol (TC) and triglyceride (TG), and abnormal decrease of high density lipoprotein lead to lipid metabolism disorder [3]. Targeting these early defects offer the most cost-effective strategy to arrest diabetic progression.

Accumulating evidence positions chronic, low-grade inflammation as a central driver of PDM-associated insulin resistance (IR) [4]. As early as 1995, Unger et al. found that the central link in the onset of diabetes is the chronic inflammatory response of target tissues [5]. In diabetes mellitus, the body often exhibits a chronic low-grade inflammatory state. It is mainly manifested as the activation of NLRP3 inflammasome, IL-1β and other inflammatory factors [6], among which NLRP3 inflammasome is the most direct receptor for inflammatory signals and a key trigger factor for inflammation [7,8]. MCC950 is highly specific for blocking NLRP3 inflammasome, which can inhibit NLRP3 and relieve the inflammatory state [9]. However, the effect of MCC950 on inflammasome factors and glucose homeostasis in PDM remains unclear. Moreover, growing studies provide exact evidence that aerobic exercise offers a non-pharmacological approach to relieve diabetes and related inflammation [10]. Studies have shown that aerobic exercise can reduce inflammation, ameliorate insulin sensitivity, and participate in the regulation of glucose homeostasis by inhibiting the NLRP3 inflammasome and promoting the PI3K/Akt signaling [6]. In addition, aerobic exercise offers a non-pharmacological approach to managing inflammation by the inhibition of NLRP3 inflammasome, which can lead to alleviate IR and liver damage in older PDM [11]. Our previous research further revealed that aerobic exercise was an effective way to alleviate blood glucose and IR in prediabetes, particularly through its impact on the NLRP3 inflammasome [12]. In summary, aerobic exercise can alleviate diabetes and PDM by inhibiting NLRP3 inflammasome. However, the underlying molecular mechanisms are still elusive.

Irisin has been reported to have important potential to reduce gluconeogenesis, and enhanced glucose utilization could make it be an important role in diabetes management [13]. Research has indicated that irisin can alleviate its related diseases by reducing the NLRP3 inflammasome [14,15]. Regular exercise training is a beneficial practice for increasing irisin secretion. In rodents, aerobic exercise has been proven to induce the increased secretion of irisin in muscle tissue [16,17] and serum, and up-regulate the irisin precursor *FNDC5* expression [18]. In healthy people, *FNDC5* mRNA expression in skeletal muscle and serum irisin are also elevated after aerobic training [19]. Nevertheless, the role of irisin signaling in the improvement of prediabetes-induced blood glucose imbalance in PDM mice and its molecular mechanism remain unclear.

Skeletal muscle plays an important role in the systemic blood glucose balance [20]. Studies have shown that the regulation of systemic glucose homeostasis primarily depends on skeletal muscle’s sensitivity to insulin and its capacity to store glycogen [21]. Therefore, we focus on irisin/NLRP3 signaling in skeletal muscle of mice with PDM in response to aerobic exercise intervention. In this study, we established the PDM mice model and applied aerobic exercise intervention to investigate: i) the relationship among the irisin/NLRP3 signaling, skeletal muscle inflammation and glucose homeostasis, and ii) changes of glucose and lipid metabolism in PDM mice after aerobic exercise intervention and its potential mechanism. Our findings are expected to provide mechanistic insight into exercise prescription and identify irisin-NLRP3 as a potential therapeutic target for PDM management.

## Research design and methods

### General protocol

**Fig 1:**
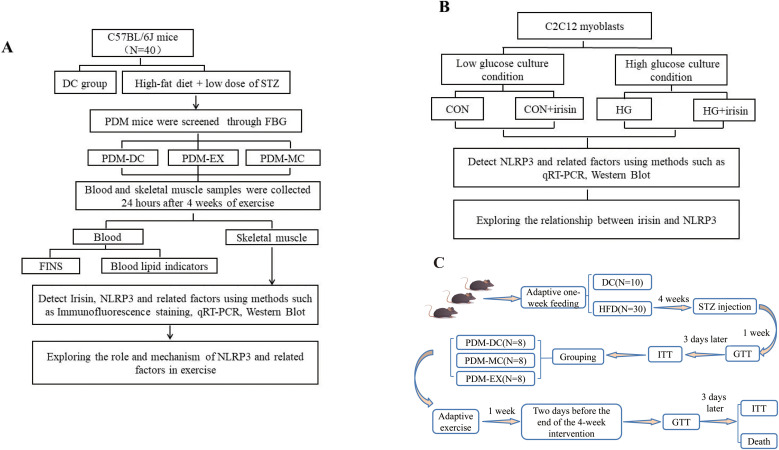
Flow chart. (A. Research flowchart of the animal experiment. B. Research flowchart of the cell experiment. C. Detailed implementation protocol of the animal experiment. Abbreviation: DC (Common diet group); STZ (Streptozotocin); PDM (Pre-diabetes mellitus); FBG (Fasting blood glucose); PDM-DC (PDM control group); PDM-MC (PDM plus MCC950 group); PDM-EX (PDM plus exercise group); FINS (fasting insulin); CON (Low glucose condition); HG (High glucose condition); HFD (high-fat diet group); GTT (Glucose Tolerance Test); ITT (Insulin Tolerance Test)).

The experiment consisted of two parts: an animal study and a cell study. The animal study aimed to investigate whether aerobic exercise alleviates pre-diabetes-induced blood-glucose dysregulation through irisin-mediated suppression of the NLRP3 inflammasome in skeletal muscle. The cell experiment was designed primarily to explore the relationship between irisin and NLRP3, and details are shown in Fig 1A and 1B.

### Experimental animals

Forty C57BL/6J mice were purchased from the Wuhan Animal Research Center. Mice were divided into the common diet group (DC group, N = 10) and high-fat diet group (HFD group, N = 30) after a week of adaptation. Maintenance diet was given in the DC group (11001, 2.37 kcal/g, Boaigang, Beijing, China). The high-fat diet was used in the HFD group (12492M, 5.24 kcal/g, Boaigang, Beijing, China). The approval number of the study is No. YZLL2022−009. All surgery was performed under Avertin injection anesthesia, and all efforts were made to minimize suffering.

### PDM mice model was established successfully

In this study, after 4 weeks of high-fat feeding, HFD mice were given a one-time intraperitoneal injection of a low dose of streptozotocin (STZ) of 110 mg/kg. After the blood glucose stabilized, the mice were fasted for 8 hours and tail-tip blood samples were collected. Fasting blood glucose (FBG) was measured using a glucometer. If the FBG of the mice was all 9.0 < FBG < 13.8 mmol/L [22], PDM mice were considered to be successfully induced. The FBG was higher obviously in the PDM group (12.68 ± 1.27 mmol/L) than that in the DC group (4.98 ± 0.68 mmol/L) (*P* < 0.01) after high-fat combined with STZ injection. A total of 24 mice met the PDM modeling criteria. Then, the PDM mice were further divided into: the PDM control group (PDM-DC group, N = 8), the PDM plus exercise group (PDM-EX group, N = 8), and the PDM plus MCC950 group (PDM-MC group, N = 8), the three groups were given the injection of equal volume of physiological saline. (As shown in Fig 1C).

### Treadmill exercise

The experiment adopted the moderate-low-intensity treadmill exercise intervention for 4 weeks. The intensity of the exercise intervention was carefully calibrated and refined based on the protocol established by Bobinski et al. [23]. PDM-EX group was treated with the adaptive treadmill exercise, and the exercise intensity was 10 m/min in the first 3 d, and 12 m/min in the second 2 d, 30 min a day for 5 days. The formal intervention time of PDM-EX group was 4 weeks, and the intensity of exercise was 12 m/min, 60 min/ d and 5 d/week. Training time is from 5:00–6:00 pm. The PDM mice maintained a high-fat diet during the intervention period.

### Inhibitor intervention

MCC950 was selected as the inhibitor of NLRP3. PDM-MC group mice were intraperitoneally injected with MCC950 (Selleck, S7809) at an experimental dose of 10 mg/kg body weight according to Wang et al. (2022) [24], and injection volume of 10 ml/kg, 5 times/week. The intervention period was 4 weeks.

### Glucose Tolerance Test (GTT) and Insulin Tolerance Test (ITT)

The mice were gavaged with 20% glucose solution (1 g/kg) after a 12-h fast, following the blood glucose was detected at 0, 30, 60, 90, and 120 min. ITT was determined 3 days after detecting GTT. After fasting for 6 h, the mice were intraperitoneally injected with insulin solution (1 IU/kg), blood glucose was measured at 0, 30, 60, 90, and 120 min followed.

### Dilution of irisin

Dissolve 10 µg of irisin powder (MedChemExpress, HY-P70664) in 100 µl of sterile dd H₂O to prepare a 100 µg/ml stock solution. Take 2 µl of the stock solution, add 48 µl of sterile dd H₂O, mix well, and dilute to a working solution of 4 ng/µl. Take 5 µl of the working solution, add it to 2 ml of cell culture medium, mix well, and obtain a final irisin concentration of 10 ng/ml.

### Culture and grouping of C2C12 myoblasts

To test irisin signaling under low *vs.* high glucose conditions, C2C12 myoblasts were digested by pancreatic enzymes and inoculated into six-well plates using DMEM medium (low glucose, containing 10% fetal bovine serum) and cultured at 37 °C and 5% CO2 for 24 hours. When the cell density grew to about 80%, the original culture medium was removed and divided into four groups, the culture medium was: ① DMEM (low glucose, containing 10% fetal bovine serum), referred to as CON condition. ② DMEM (low glucose, containing 10% fetal bovine serum) plus 10 ng/ml irisin, referred to as CON + irisin condition. ③ DMEM (high glucose, containing 10% fetal bovine serum), referred to as HG condition. ④ DMEM (high glucose, containing 10% fetal bovine serum) plus 10 ng/ml irisin, referred to as HG + irisin condition.

### Tissue samples

24 hours after the last treadmill exercise, a 12-h fast followed. Mice were anesthetized with isoflurane for 5 min, and blood was collected from the retro-orbital venous plexus. They were then euthanized by intraperitoneal injection of Averlin (20 μl/g, Dowobio, DW3101). The blood samples were left at room temperature for 30 min before centrifugation. The quadriceps muscle was quickly removed, part of the muscle was placed in muscle fixation solution (for morphological detection, Pinofei Biotechnology Company, P0003), part of the muscle was put into liquid nitrogen, and then transferred to the refrigerator at −80 °C for storage to be measured.

### Biochemical index detection

Before the test, the serum stored at −80 °C was thawed, and the serum levels of fasting insulin (FINS) (Solarbio Company, YZ-140633) was detected by ELISA. High density lipoprotein cholesterol (HDL-C), TC, TG and low density lipoprotein cholesterol (LDL-C) were analyzed by automatic biochemical analyzer (Hitachi 7600). To calculate IR, we used the homeostatic model assessment for IR (HOMA-IR), the formula is: HOMA-IR = FINS×FBG/22.5.

### Hematoxylin and eosin (H&E) staining

Muscle sections were subjected to dehydration, transparenting, paraffin embedding, slicing, baking, and dewaxing. Muscle section were stained with H&E staining solution 1 (main component: hematoxylin), H&E staining solution 2 (main component: differentiation solution) and H&E staining solution 3 (main component: reverting blue solution), respectively. The slices were immersed in 85% ethanol, 95% ethanol, H&E staining solution 4 (the main component is eosin), anhydrous ethanol I, anhydrous ethanol II, anhydrous ethanol III, n-butanol, xylene I, xylene II, and sealed with neutral gum. Observation with Olympus BX51 microscope (Olympus, Tokyo, Japan).

### Immunofluorescence staining

Paraffin sections (4 µm) were baked at 60 °C for 30 min, deparaffinized in xylene (two changes, 10 min each) and rehydrated through graded ethanols (100%, 100%, 95%, 85%, 75%, 5, 5, 3, 3, 3 min, respectively). Slides were then washed three times in PBS (5 min each). Antigen retrieval was performed by immersing the sections in 10 mM sodium citrate buffer (pH 6.0) and heating in a pressure cooker at full pressure for 2 min; the buffer was allowed to cool to room temperature. After three PBS washes (5 min each), non-specific binding was blocked with 10% normal goat serum for 30 min at room temperature. Excess serum was tipped off and the sections were incubated overnight at 4 °C with rabbit anti-NLRP3 primary antibody (1:200; Abways, CY5651) diluted in antibody diluent. Following three washes in PBST (5 min each), alexa-Fluor-conjugated secondary antibody (diluted in PBST) was applied for 1 h at room temperature in the dark. Sections were again washed three times in PBST (5 min each), counterstained with DAPI (1 µg/ml, 5 min, dark) and given three final PBS rinses (5 min each). Slides were mounted with fluorescence-compatible mounting medium and stored at 4 °C in the dark. Images were acquired on an Olympus BX51 fluorescence microscope (Olympus, Tokyo, Japan).

### Quantitative reverse transcription polymerase chain reaction (qRT-PCR)

qRT-PCR was employed to detect the mRNA expression of NLRP3 and other inflammation-related indicators. TRIzol (Vazyme, R411-01) extracted total RNA from muscle tissue or C2C12 myoblasts. The extracted RNA was then purified to remove any impurities. Reverse transcription kit (TAKARA, RR047A) was used to transcribe purified RNA into cDNA. Fluorescent quantitative kit (TAKARA, RR420A) and fluorescent quantitative PCR instrument (ThermoFisher Scientific, ABI7500) were used for detection. The primer of target geneand (Tianyi Huiyuan) and internal reference (AuGCT) were added. β-actin was used as the control. Table 1 shows the sequence of primers used. CT values were read using fluorescent quantitative PCR (ThermoFisher Scientific, ABI7500) software.

**Table 1:** Sequences of primers used for qRT-PCR.

Target gene		Genetic sequence
FNDC5	Forward primer 5’-3’	AGGCTGCGTCTGCTTCG
	Reverse primer 5’-3’	CACTGGCTGGGCTCTGT
NLRP3	Forward primer 5’-3’	GTTCTTCGCTGCTATGT
	Reverse primer 5’-3’	TTCAAACTTGCCGTAAT
IL-18	Forward primer 5’-3’	CAGGCCTGACATCTTCTGCAA
	Reverse primer 5’-3’	CTGACATGGCAGCCATTGT
IL-1β	Forward primer 5’-3’	GCAGGCAGTATCACTCATTG
	Reverse primer 5’-3’	GCTTTTTTGTTGTTCATCTC
β-actin	Forward primer 5’-3’	TGGTGGGAATGGGTCAGAA
	Reverse primer 5’-3’	ATGGCTGGGGTGTTGAAGG
GAPDH	Forward primer 5’-3’	CTCAGGAGAGTGTTTCCTCGT
	Reverse primer 5’-3’	ATGAAGGGGTCGTTGATGGC

### Western blot

Sixty milligrams of skeletal muscle were transferred into a 2-mL QSP tube, minced on ice, and mixed with 600 µL ice-cold lysis buffer prepared at a ratio of 100:1:2 (RIPA: PMSF: protease and phosphatase inhibitor cocktail). After centrifugation, the supernatants were collected and the proteins were separated by SDS-PAGE, then electro-transferred onto PVDF membranes using a wet-transfer system. The membranes were blocked with 5% (w/v) BSA in TBST for 1 h at room temperature and incubated overnight at 4 °C with the following primary antibodies: β-actin (Cell Signaling, 4970S) at 1:1000, NLRP3 (Wanleibio, WL02635) at 1:1000, IL-1β (Proteintech, 26048–1-AP) at 1:1000, and IL-18 (Proteintech, 10663–1-AP) at 1:1000. After three 5-min washes in TBST, the membranes were incubated for 1 h at room temperature with the appropriate HRP-conjugated secondary antibodies (diluted 1:1000 in 5% BSA). Following three additional TBST washes, immunoreactive bands were visualized with an enhanced chemiluminescence system and quantified by densitometry.

### Statistical analysis

SPSS 13.0 was employed for all statistical analyses. The sample size calculation was based on an expected effect size of d = 1.2, with 80% power and a two-sided alpha of 0.05, indicating a minimum of n = 8 animals per group. Normality of data distribution was assessed using the Shapiro–Wilk test, and homogeneity of variances was verified with Levene’s test prior to parametric analysis. The comparison between the two groups was performed using an independent sample T test. For groups of three or more, one-way analysis of variance (ANOVA) was used to analyze the differences. The intra-group comparison performed the paired sample T test. *P* < 0.05 was typically considered statistically significant. Western blot results were analyzed using Image J software, and histograms are plotted using the GraphPad Prism 8.0 software.

## Results

### PDM model was successfully established

**Fig 2:**
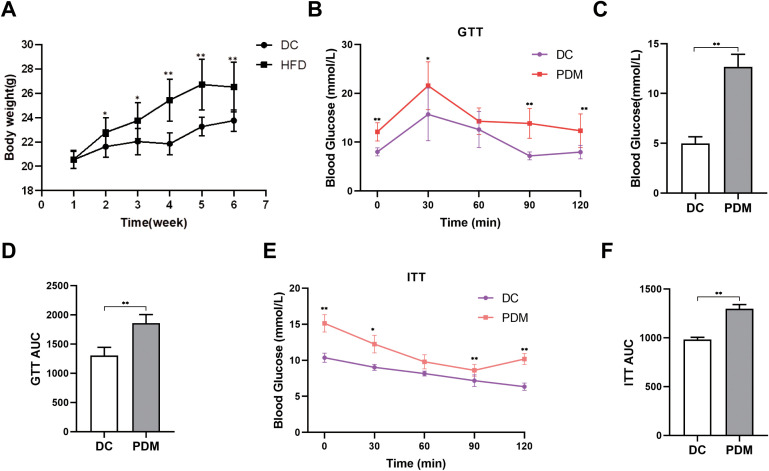
Changes of metabolic indexes in the PDM group and DC group. (A. Changes in body weight during the high-fat diet. B. Curve of glucose tolerance test in the PDM group and DC group. C. FBG in the PDM group and DC group. D. AUC of the glucose tolerance. E. Curve of insulin tolerance test in the PDM group and DC group. F. AUC of the insulin tolerance. ^*^P < 0.05, ^**^P < 0.01 *vs*. DC.).

The experimental data showed that there was no statistical significance in body weight between the DC and HFD groups in the first week. From the second week, the HFD group had significant weight gain after high-fat feeding (*P* < 0.05 or *P* < 0.01) (Fig 2A). The blood glucose level in the GTT test showed a trend of first rising and then decreasing, and the peak value of blood glucose appeared around 30 min and then decreased. The PDM group showed higher blood glucose levels compared to the DC group at each of the measured time points (*P* < 0.05 or *P* < 0.01) after gavage with glucose solution or injection of insulin solution (Fig 2B and 2E). Moreover, a higher area under the curve (AUC) for both glucose and insulin tolerance tests in the PDM group compared to the DC group (*P* < 0.01) (Fig 2D and 2F). These results all indicate that FBG was elevated, and glucose tolerance and insulin tolerance were impaired in PDM group (*P* < 0.01) (Fig 2C), illustrating that the PDM model was constructed successfully.

### Benefit of aerobic exercise on body weight and glycolipid metabolism in mice with PDM

**Fig 3:**
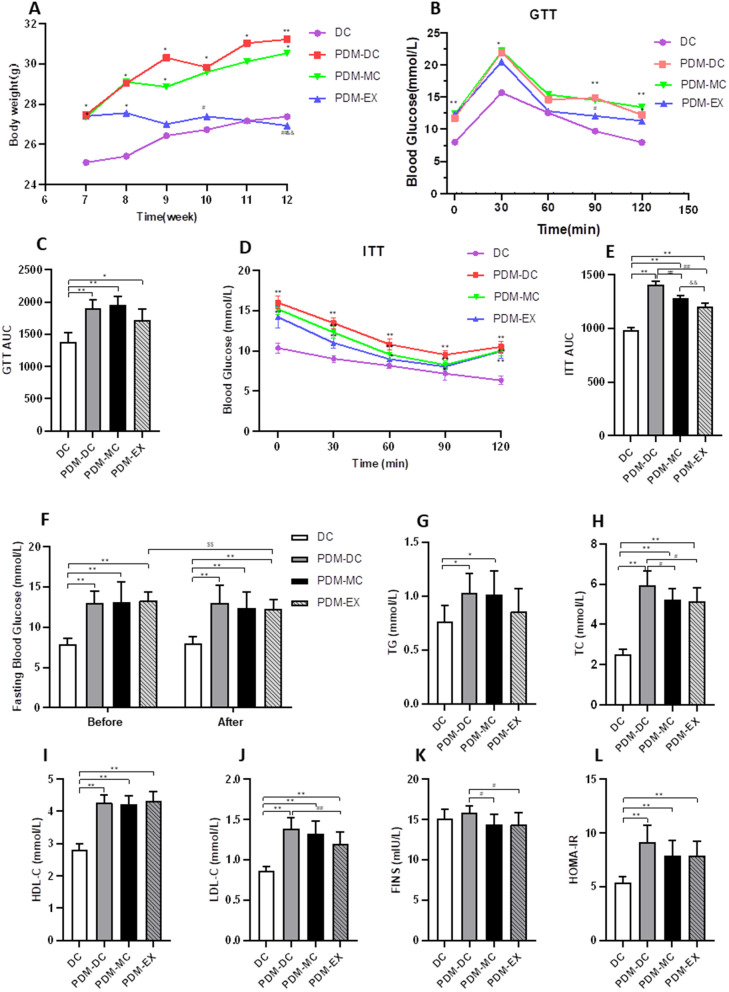
Changes in body weight and glycolipid metabolism in different groups. (A. Effects of aerobic exercise on body weight. B. Curve of glucose tolerance test in different groups. C. Area under the curve of the glucose tolerance. D. Curve of insulin tolerance test in different groups. E. Area under the curve of the insulin tolerance. F. Fasting blood glucose in different groups before and after intervention. G. Changes of TG in different groups. H. Changes of TC in different groups. I. Changes of HDL-C in different groups. J. Changes of LDL-C in different groups. K. Changes of FINS in different groups. L. Changes of HOMA-IR in different groups. ^*^P < 0.05, ^**^P < 0.01 *vs*. DC; ^#^*P* < 0.05, ^##^*P* < 0.01 *vs*. PDM-DC;^$$^ *P* < 0.01 *vs*. before.).

The body weight and FBG of the PDM group were elevated obviously than that of the DC group (*P* < 0.05) (Fig 3A and 3F). We found that the body weight in the PDM-EX group decreased compared with both the PDM-DC and PDM-MC groups (*P* < 0.01) (Fig 3A). Furthermore, it was also found that the PDM-EX group exhibited lower levels of blood glucose when compared to the PDM-DC group at all measured time points after gavage with glucose solution or injection in insulin solution (Fig 3B and 3D). The AUC of insulin tolerance (*P* < 0.01) (Fig 3E) were significantly lower than that in the PDM-DC group. As shown in Fig 3F, after 4 weeks of intervention, FBG in the PDM-EX group was declined than that in the PDM-DC group (*P* < 0.01).

Moreover, to investigate the changes of lipid metabolism after intervention, we examined relevant factors. TG, TC, HDL-C, LDL-C and HOMA-IR in the PDM-DC group were significantly elevated compared to those in the DC group (*P* < 0.05 or *P* < 0.01), demonstrating that these lipid metabolism indexes of the PDM group increased than those in the DC group. Compared with the PDM-DC group, TC, LDL-C and FINS in the PDM-EX group and PDM-MC group all decreased after 4 weeks of intervention, and the decrease was more obvious in the PDM-EX group (*P* < 0.05 or *P* < 0.01) (Fig 3G–3L). This result suggested that aerobic exercise could improve serum insulin concentration, insulin sensitivity and lipid metabolism in PDM mice. Additionally, compared with the PDM-DC group, the PDM-EX group showed no significant reductions in either TG or HOMA-IR (*P* > 0.05), possibly related to the fact that the blood glucose of PDM has not reached the diabetes standard or the insufficient intervention time.

### Aerobic exercise antagonizes tissue morphological damage caused by PDM

**Fig 4:**
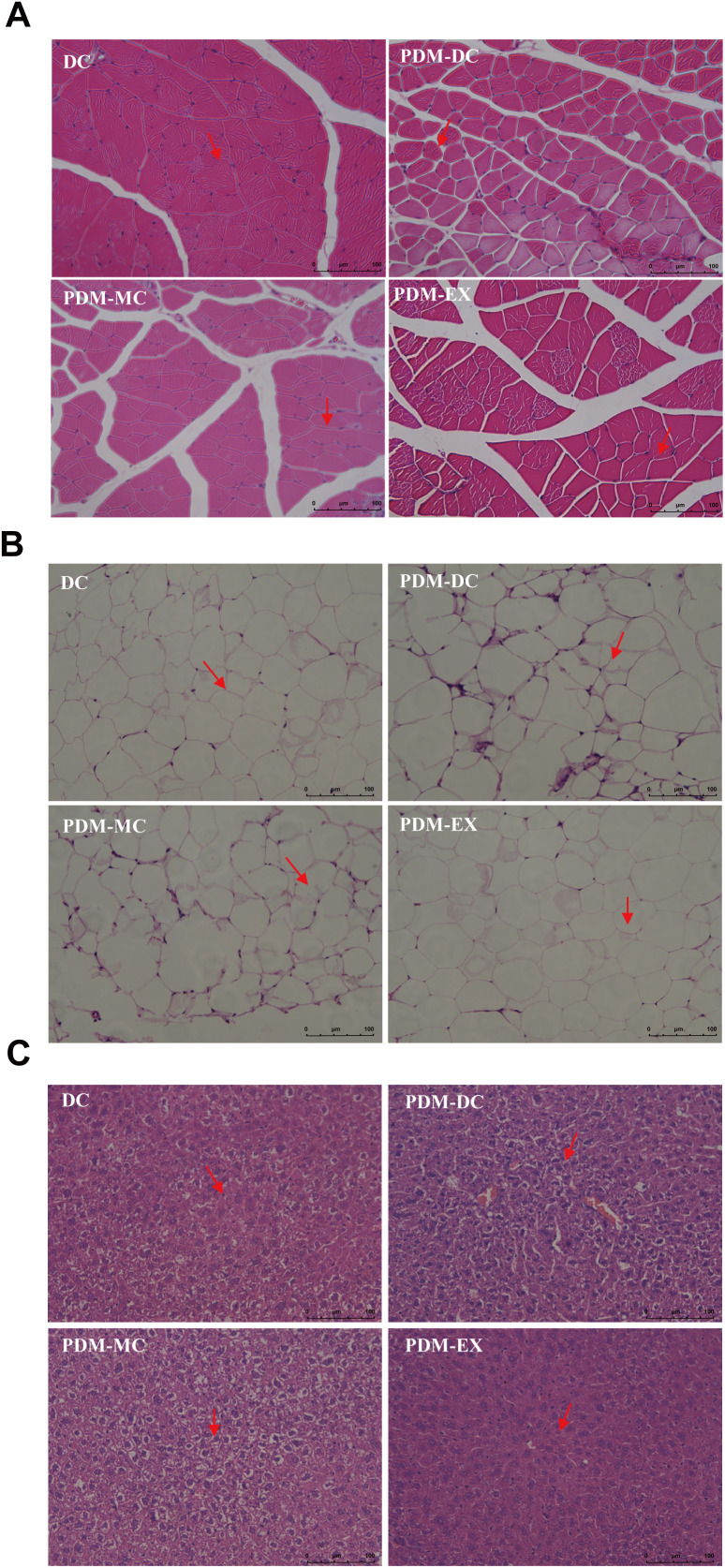
Morphological changes of skeletal muscle, fat and liver in each group. (200 × , Scale bars, 100 µm; N = 3 for each group). (A. Morphological changes of skeletal muscle in each group. B. Morphological changes of fat in each group. C. Morphological changes of liver in each group. The red arrows indicate mainly the arrangement of the cells.).

As illustrated in Fig 4A, H&E staining results indicated that the myocytes were arranged loosely, the tissue morphology spread was poor, the muscle fiber gap was larger, the arrangement was disordered in the PDM-DC group compared to the DC group. The muscle fiber space in the PDM-EX and PDM-MC groups became smaller, the cavity in the muscle fiber was less, and the cell structure tended to be completed when compared with the PDM-DC group. Similar to MCC950, aerobic exercise has been suggested to render the skeletal muscle remodeling of PDM mice.

Since adipose tissue and liver are also involved in glycolipid metabolism, we chose adipose tissue and liver to observe. As shown in Fig 4B, the fat cells were large and arranged loosely and irregularly in the PDM-DC group when compared with the DC group. Compared with the PDM-DC group, the fat cells were orderly and regularly in the PDM-EX and PDM-MC groups. As illustrated in Fig 4C, the liver damage area of DC group was smaller than that of the PDM-DC group. The liver damage areas of the PDM-EX and PDM-MC groups were smaller than those of the PDM-DC group.

### Aerobic exercise increases FNDC5/irisin expressions and inhibits the activation of NLRP3 and its related factors in the skeletal muscle of PDM mice

To further study the mechanism of aerobic exercise on PDM. The expression of irisin, NLRP3 and its related factors were detected by qRT-PCR, western blot and immunofluorescent staining.

**Fig 5:**
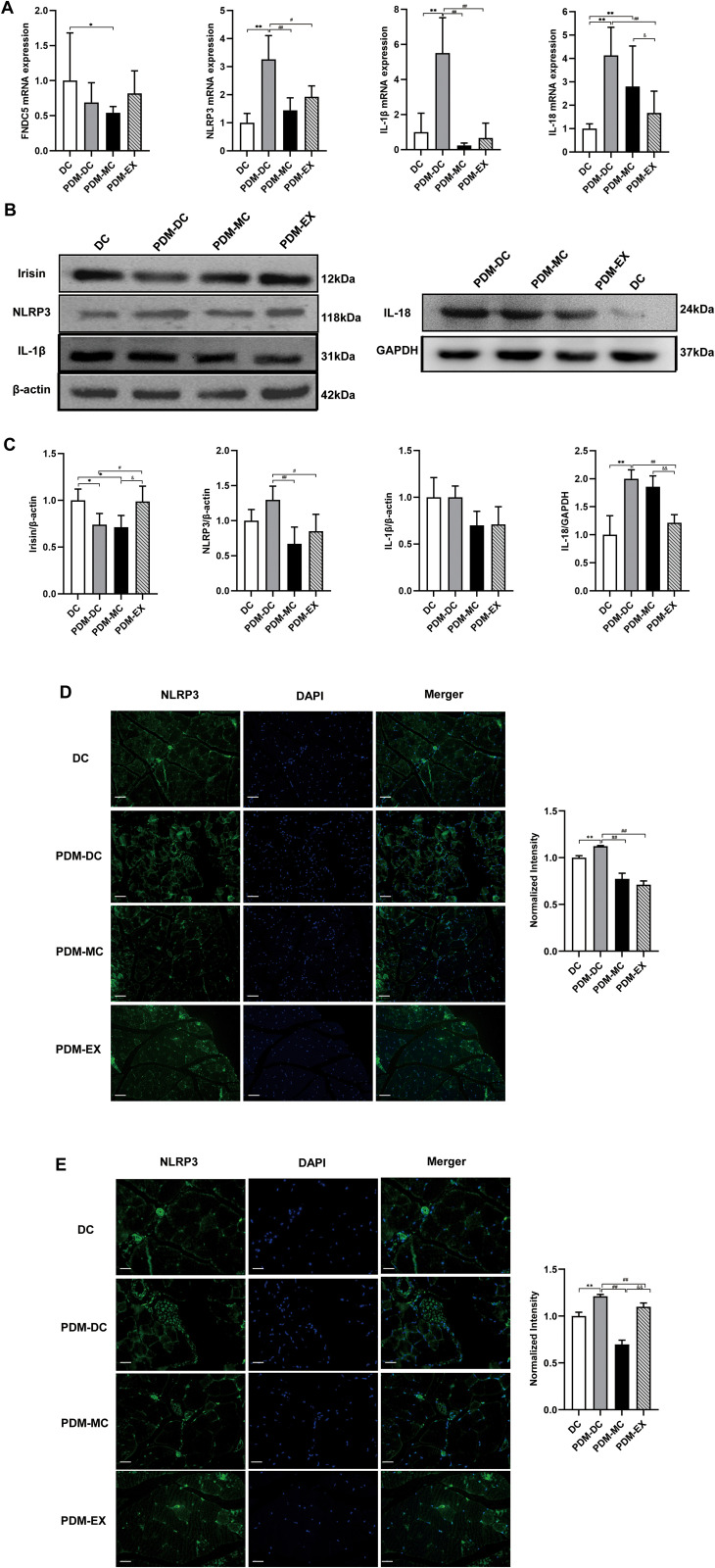
Effects of aerobic exercise on the expression levels of NLRP3 and its related factors in the skeletal muscle of mice. (A. Real-time qPCR analysis was performed to detect the mRNA levels of *FNDC5* and its related factors in skeletal muscle. B. Western blot analysis was performed to detect the protein expression levels of Irisin and its related factors in the skeletal muscle of mice. C. Quantitative analysis of the relative expression levels of Irisin protein and its related factors in Fig.B. D. Immunofluorescence staining of the skeletal muscle tissue (200 × , Scale bars, 100 µm; N = 3 for each group). E. Immunofluorescence staining of skeletal muscle tissue (400 × , Scale bars, 50 µm; N = 3 for each group). The green color represents areas of NLRP3 inflammasome, and the blue color represents the nucleus. ^*^P < 0.05, ^**^P < 0.01 *vs*. DC; ^#^*P* < 0.05, ^##^*P* < 0.01 *vs*. PDM-DC; ^&^*P* < 0.05, ^&&^*P* < 0.01 *vs*. PDM-MC.).

Firstly, mRNA expression levels of *FNDC5*, NLRP3 and its related factors in PDM mice were detected. As shown in Fig 5A, the qRT-PCR results showed that compared with the DC group, *FNDC5* mRNA expression was lower, while the NLRP3, IL-1β and IL-18 mRNA levels were higher in the PDM-DC group (*P* < 0.01). The experimental data indicated that *FNDC5* mRNA expression was increased, the mRNA expression of NLRP3, IL-1β, IL-18 was lower significantly (*P* < 0.05 or *P* < 0.01) in the PDM-MC and PDM-EX groups than that in PDM-DC group after intervention.

Secondly, the western blot results confirmed that the protein expression of irisin was reduced, the NLRP3, IL-1β and IL-18 expression levels (*P* < 0.05 or *P* < 0.01) were higher in skeletal muscle of the PDM-DC group when compared with the DC group. Furthermore, the irisin protein expression was increased obviously (*P* < 0.05), the protein expression of NLRP3, IL-1β and IL-18 (*P* < 0.01) were decreased in the PDM-EX group compared to the PDM-DC group (Fig 5B and 5C).

Finally, to further confirm the NLRP3 expression and inflammatory state in the skeletal muscle tissue of PDM mice, tissue immunofluorescence staining was applied. The findings showed that there were a higher number of NLRP3-positive cells in the PDM-DC group in comparison with the DC group (Fig 5D and 5E). The distribution of green fluorescence in the DC group was more loose and less concentrated, while the PDM-DC group was more tight and dense. Moreover, the PDM-DC group exhibited a higher average fluorescence intensity compared to the DC group (*P* < 0.01), suggesting that NLRP3 protein is expressed in large quantities in the skeletal muscle tissue cells of PDM mice. We found that aerobic exercise and MCC950 can decrease the content of NLRP3 in skeletal muscle of PDM mice. Furthermore, the NLRP3-positive cells were arranged more neatly and their structure tended to be regular in the PDM-MC group and PDM-EX group when compared with the PDM-DC group.

The average fluorescence intensity of the PDM-MC group and PDM-EX group were lower significantly than that in the PDM-DC group (*P* < 0.01). These results indicated that aerobic exercise could effectively inhibit the NLRP3 expression and its related inflammatory response in the skeletal muscle of PDM mice as well as NLRP3 inhibitor (MCC950).

In a word, these consequences have manifested that aerobic exercise could enhance the *FNDC5*/irisin expression, inhibit the activation of NLRP3 and its related factors in skeletal muscle of PDM mice. However, the relationship between Irisin and NLRP3 needs to be further explored.

### Irisin inhibited the expression of NLRP3 and inflammation-related factors in C2C12 cells

**Fig 6:**
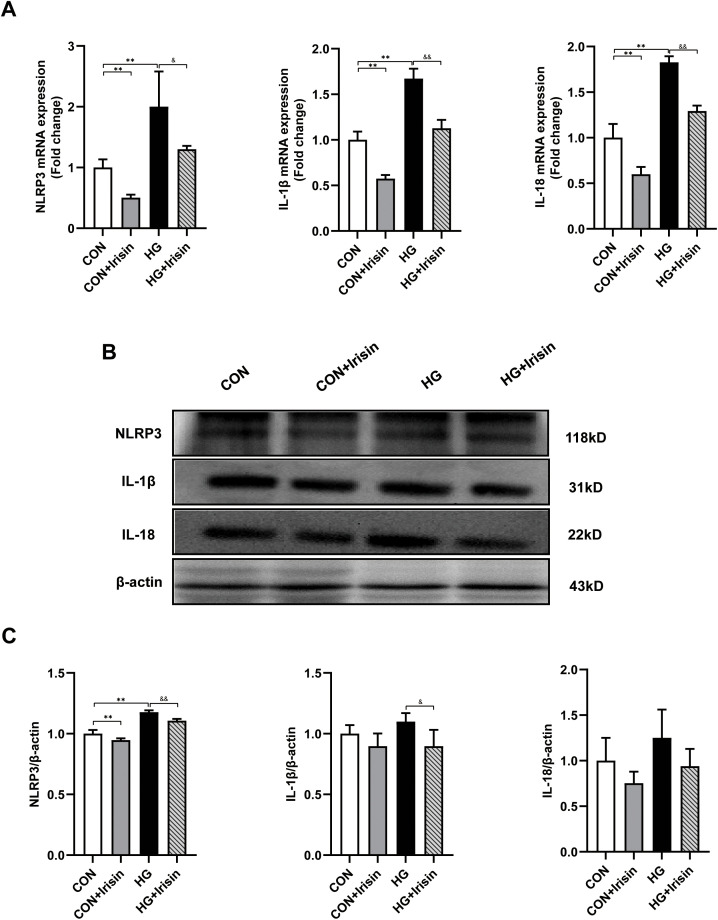
The expression of irisin, NLRP3 and inflammation-related factors in C2C12 cells. (A. Real-time qPCR analysis was performed to detect the mRNA levels of irisin, NLRP3 and inflammation-related factors in C2C12 cells. B. Western blot analysis was performed to detect the protein expression levels of irisin, NLRP3 and inflammation-related factors in the C2C12 cells. C. Quantitative analysis of the relative expression levels of irisin, NLRP3 and inflammation-related factors in Figure B. ^*^P < 0.05, ^**^P < 0.01 *vs*. CON; ^&^*P* < 0.05, ^&&^*P* < 0.01 *vs*. HG.).

To further verify the irisin/NLRP3 signal transduction in the skeletal muscle, firstly, the effect of high glucose on the NLRP3 inflammasome was examined. Next, we incubated C2C12 cells with irisin (10 ng/mL) in the absence or presence of high glucose. The qRT-PCR results found that the mRNA expression of NLRP3, IL-1β and IL-18 in the HG condition were higher compared with the CON condition (*P* < 0.01). Moreover, irisin can lower the NLRP3, IL-1β and IL-18 mRNA expression (*P* < 0.05 or *P* < 0.01) (Fig 6A). The western blot findings showed that compared with the CON condition, the protein expression levels of NLRP3 (*P* < 0.01), IL-1β and IL-18 in the HG condition were increased (Fig 6B and 6C), suggesting that high glucose could induce inflammation. Moreover, at 10 ng/mL, the NLRP3, IL-1β and IL-18 expressions had obvious decreasing trend in the CON + irisin and HG + irisin conditions (Fig 6B and 6C), indicating that irisin can block the increase of NLRP3, IL-1β and IL-18 expression induced by high glucose.

## Discussion

Skeletal muscle, which makes up about 40% of body weight [25], is the largest insulin-sensitive organ and takes a significant factor in the systemic blood glucose balance, with 80% of insulin-mediated glucose absorption completed by skeletal muscle [20]. It has been found that the regulation of systemic glucose homeostasis is mainly dependent on the skeletal muscle’s sensitivity to insulin and capacity for glycogen storage [21]. Chronic inflammation, oxidative stress, and insulin secretion disorder can lead to an imbalance of blood glucose homeostasis, decreased insulin sensitivity, and IR [26]. Chronic inflammation plays a pivotal factor in the development of diabetes, accompanied by elevated levels of NLRP3 inflammasome and other inflammatory factors. Therefore, while reducing blood glucose and IR, it is particularly critical to explore safe and effective strategies to alleviate chronic inflammation of diabetes. Here, the PDM model was established successfully, and the animal and cell experiments were applied to explore the effect and mechanism on alleviating chronic inflammation caused by diabetes.

Irisin, a novel muscle factor discovered in 2012, is hydrolyzed by *FNDC5* and is the main protein that promotes browning of white adipose tissue (WAT) and increases body energy consumption [27]. The skeletal muscle expresses and secretes the most Irisin in the body, reaching 75% of the total irisin in the circulating blood [28]. Irisin has been identified as the potential factor in metabolism and energy homeostasis, and is closely associated with metabolic diseases [29]. The present study found that the *FNDC5*/irisin expression was decreased in PDM mice, suggesting that lower *FNDC5*/irisin expression level might be the risk for PDM. The NLRP3 inflammasome is composed of sensor protein (NLRP3), adaptor protein (ASC) and effector protein (Caspase-1), and plays a significant role in various physiological processes, including inflammation, adipocyte differentiation, and insulin sensitivity [30]. NLRP3 inflammasome is capable of sensing obesity-related danger signals and is closely related to insulin signals, glucose tolerance, and IR [31,32]. Various studies have verified that the expression and activation of the NLRP3 inflammasome and related factors are believed to be involved in the occurrence and progression of IR and T2DM [6,33]. Elevated mRNA and protein expression of NLRP3 inflammasome, and IL-1β have been found in diabetic rats [34]. The data from our study also found that raised NLRP3 expression in the PDM mice. These results mirror previous findings from our group [12], stating that the NLRP3 signaling pathway made a decisive contribution to PDM in both mice models and patients. The NLRP3 inflammasome and its downstream factors IL-1β and IL-18 are considered to be critical players in the body’s inflammatory responses [35]. IL-1β and IL-18 are closely related to the occurrence and development of diabetes mellitus and are associated with metabolic homeostasis, inflammation, and IR [36,37]. A previous study showed that serum IL-1β and IL-18 concentrations increased in the PDM patients [12]. The qRT-PCR and western blot experiments demonstrated that the expression level of IL-18 in the skeletal muscle of PDM mice was significantly increased. Moreover, we found that the IL-1β mRNA expression was increased obviously, but IL-1β protein expression in PDM mice was not significantly different. However, we also found that irisin significantly blocked the increase of NLRP3, IL-1β and IL-18 expression induced by high glucose. In addition, irisin can inhibit the expression of NLRP3 inflammasome and related factors in C2C12 myoblasts. This is largely in line with the results reported by Li et al., who found that in cardiac microvascular endothelial cells exposed to high glucose, irisin significantly suppressed NLRP3 inflammasome assembly and down-regulated NLRP3, IL-1β and IL-18 expression at both the mRNA and protein levels, and that overexpression of NLRP3 reversed the protective effects of irisin [38].

Aerobic exercise has been proven to antagonize inflammatory response, but the molecular mechanism is still largely unknown. The current findings have shown that the irisin expression in the skeletal muscle increased significantly after aerobic exercise intervention in PDM mice. Clinical trials and animal studies have demonstated that aerobic exercise can increase the irisin secretion and the *FNDC5* expression [19,27]. Bostrom et al. indicated that the serum irisin content in mice were increased by 65% after three weeks of free running [27]. Kim et al. reported that irisin levels in the muscles of diabetic rats were enhanced significantly after resistance training [16]. Lee et al. showed that the *FNDC5* mRNA expression in skeletal muscle and serum irisin content raised obviously in healthy men after aerobic training [19]. To sum up, aerobic exercise can increase the irisin expression in PDM mice. Studies identified that inhibiting the NLRP3 inflammasome activation could alleviate diabetes and diabetes complications [39,40]. Our early research reported that aerobic exercise can significantly ameliorate the chronic inflammatory state in PDM people, accompanied by the reduction of NLRP3 [12]. In animal models, our data suggested that the decrease in NLRP3 expression in PDM skeletal muscle following aerobic exercise. Multiple studies have indicated that aerobic exercise plays a pivotal role in regulating the NLRP3 inflammasome [41,42]. Wang et al. found that 4 weeks of aerobic endurance training significantly inhibited the activity of NLRP3 inflammasome in the depression mice [43]. Yang et al. showed that aerobic exercise has the potential to alleviate non-alcoholic steatohepatitis through the suppression of the NLRP3 inflammasome [41]. It is reported that aerobic exercise can also affect NLRP3 inflammasome-related proteins [44], and inhibit the NLRP3 inflammasome activation in adipose tissue [42]. Consistent with these previous findings, 4 weeks of aerobic exercise could enable downregulation of NLRP3 in PDM mice. Cumulating evidence has suggested that the expression of IL-1β and IL-18 were prohibited by aerobic exercise, thereby improving the inflammatory state [45,46]. The findings by Lee et al. demonstrated that NLRP3 mRNA expression was decreased in adipose tissue, accompanied by a decline in IL-1β and IL-18 expression in plasma of trained obese mice [45]. Tomeleri et al. showed that the potential effects of 12-week resistance exercise in older women with T2DM, particularly in reducing the expression of IL-1β and IL-18 [46]. In the current study, we found that IL-18 was also decreased after aerobic exercise. However, aerobic exercise could reduce the IL-1β mRNA expression in PDM, but did not significantly decrease the IL-1β protein expression. On the one hand, it is possible caused by the insufficient intervention time; on the other hand, IL-1β is a secretory protein. The possible reason need to be further explored in follow-up studies. As mentioned above, aerobic exercise might antagonize the inflammatory reaction by activating irisin, and then further inhibiting the expression of NLRP3 and IL-18 of PDM mice.

**Fig 7:**
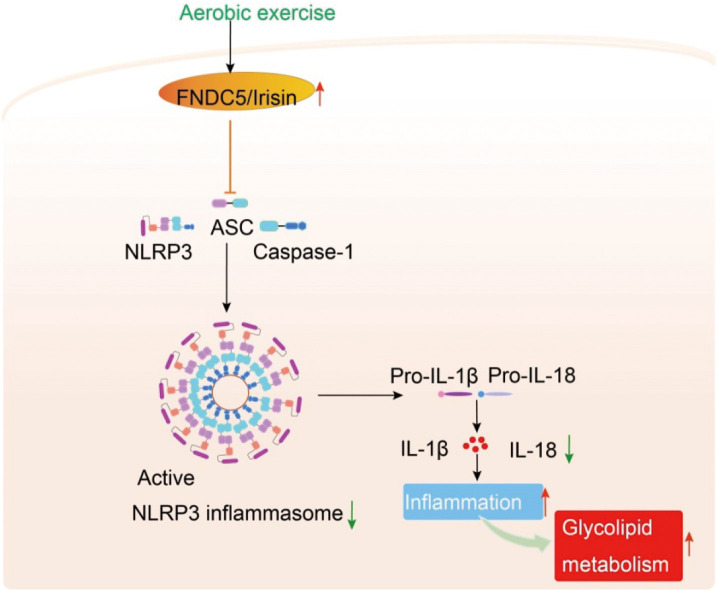
Potential mechanism of aerobic exercise in regulating glycolipid metabolism of PDM mice. (Aerobic exercise can increase the level of irisin in the skeletal muscle, thus inhibiting the expression of NLRP3 inflammasome and IL-18, ameliorating the inflammation state and glycolipid metabolism. The red arrows represent that it can be alleviated by aerobic exercise, and the green arrows indicate that its expression can be downregulated by aerobic exercise.).

Increasing evidence has suggested that irisin can reduce inflammation by inhibiting the NLRP3 activation [47]. Deng et al. further found that irisin can alleviate inflammation and endothelial dysfunction in vascular complications of diabetes by blocking ROS-NLRP3 inflammatory signaling [48]. Irisin has also been revealed to protect against liver damage by arresting NLRP3 inflammasome activation [49]. However, the mechanism of irisin in aerobic exercise alleviated glycolipid metabolism is unclear. In this study, we found that aerobic exercise could reduce FBG, TC, LDL-C, and FINS of PDM, indicating that aerobic exercise can alleviate glycolipid metabolism. As summarized in Fig 7, the skeletal irisin level was upregulated, and the downstream inflammatory factors NLRP3 and IL-18 were downregulated when PDM mice performed with aerobic exercise, thereby alleviating glycolipid metabolism, including reduction of body weight and fasting glucose, improvement of glycolipid metabolism. In summary, the results suggested that aerobic exercise might alleviate glycolipid metabolism in PDM mice by activating irisin, and then further inhibiting the expression of NLRP3 and IL-18 in skeletal muscle, further confirming that regulation of irisin/NLRP3 signal transduction is the basis of aerobic exercise to maintain blood glucose homeostasis.

## Limitations

The mice underwent treadmill exercise at a fixed intensity, which may lead to rapid adaptation and consequently diminish the training benefits. Follow-up studies that measure a VO_2_ Max to adjust the intensity of exercise may be even more effective. If the exercise + inhibitor group is added in animal experiments, it will be more accurate to understand and verify the effect of exercise on the NLRP3 inflammasome and related signaling pathways. In addition, the specific targets through which irisin and NLRP3 signals affect insulin signal transduction pathways have not been involved in this experiment, which should be further studied and discussed in subsequent studies.

## Conclusion

In conclusion, our study confirmed that the expression of NLRP3 and IL-18 were elevated, while irisin was reduced in PDM mice. Importantly, aerobic exercise was unveiled to activate irisin, thereby inhibiting NLRP3/IL-18 and its subsequent inflammatory response and glycolipid metabolism, which provides effective approaches for the management of diabetes by using aerobic exercise intervention.
